# Epigenetic Effects of Gut Metabolites: Exploring the Path of Dietary Prevention of Type 1 Diabetes

**DOI:** 10.3389/fnut.2020.563605

**Published:** 2020-09-24

**Authors:** Ahmad Al Theyab, Turki Almutairi, Abdulla M. Al-Suwaidi, Ghizlane Bendriss, Clare McVeigh, Ali Chaari

**Affiliations:** Premedical Division, Weill Cornell Medicine Qatar, Doha, Qatar

**Keywords:** type 1 diabetes, gut microbiota, epigenetics, diet, prebiotics, probiotics

## Abstract

Type 1 diabetes (T1D) has increased over the past half century and has now become the second most frequent autoimmune disease in childhood and one of major public health concern worldwide. Evidence suggests that modern lifestyles and rapid environmental changes are driving factors that underlie this increase. The integration of these two factors brings about changes in food intake. This, in turn, alters epigenetic regulations of the genome and intestinal microbiota composition, which may ultimately play a role in pathogenesis of T1D. Recent evidence shows that dysbiosis of the gut microbiota is closely associated with T1D and that a dietary intervention can influence epigenetic changes associated with this disease and may modify gene expression patterns through epigenetic mechanisms. In this review focus on how a diet can shape the gut microbiome, its effect on the epigenome in T1D, and the future of T1D management by microbiome therapy.

## Introduction

Type 1 Diabetes (T1D) is one of the two major autoimmune disorders in infants and adolescents. The T1D prevalence of 1:300 is increasing over the world, representing 5% to 10% of all diabetes mellitus cases (1). T1D is a chronic endocrine and metabolic disorder of carbohydrate metabolism, characterized by the autoimmune T cell-mediated damage of pancreatic β-cells (2). T1D results from the progressive loss of insulin producing β-cells in the islets of Langerhans in the pancreas, commonly in genetically predisposed individuals (3). Both innate and adaptive immune responses are involved in the destruction of beta cells, and insulin therapy becomes mandatory when 80% of the cells are affected (4). It is important to note the contribution of defects in immune regulation and genetic susceptibility that provides insight into the pathogenesis of the disease, which remains largely misunderstood (5). Literature has shown that there are substantial genetic and environmental influences on the onset of type 1 diabetes. In fact, different factors are involved in the development of T1D, including the genome, the gut microbiota and diet (6–8).

Furthermore, various studies have shown that macronutrients and micronutrients and their bioactive components, as well as probiotics, could prevent and manage diabetes mellitus and its complications by a variety of mechanisms. These range from the modulation of insulin secretion and action, to the protection of the β-cells, decrease of inflammation, and the modulation of gut microbiota (6, 9–11). In recent years, the involvement of the gut microbiota in the pathogenesis of several diseases and its role in the management of these diseases has gained much attention (12–18). Today, it is known that the gut microbiota, which refers to all microorganisms inhabiting the gut, is involved in many pathologies and targeting the gut microbiota has become a novel therapeutic strategy (19).

There are about 500–1,000 species in the gut microbiota of humans, with ~100 trillion bacterial cells in the adult intestine (20, 21). Gut microbiota is defined as the environment of commensal microorganisms (bacteria, fungi, viruses, protozoa) residing within the human gastrointestinal tract, interacting with host metabolism, immunity, and other vital physiological functions (22). Four phyla of bacteria represent the major component of the gut microbiota in the gastrointestinal tract; they are *Bacteroidetes, Firmicutes, Proteobacteria*, and *Actinobacteria* (23). *Bacteroidetes* and *Firmicutes* constitute the most abundant phyla in the adult intestine, while *Actinobacteria are* the most abundant phyla in breast fed infants (24, 25). Recent data have demonstrated that an alteration or aggravated dysbiosis of intestinal microbiota is implicated in T1D onset in European, American, and Mexican children (26). It has been shown that the diversity of gut microbiota is significantly reduced in patients with T1D, which substantiates the hypothesis that reduced microbiota diversity exacerbates disease pathogenesis (23). One hypothesis is that altered microbiota composition in infancy introduces perturbations in immune system development by promoting intestinal permeability and inflammation (27). Modulating the interface between diet and commensal microorganisms has been shown to correlate with the pathogenesis of T1D, and its onset in genetically susceptible individuals; especially by regulating the epigenetic mechanisms and the abundance of specific bacterial species (*Bifidobacteria, Firmicutes*, etc.) since they relate to T cell regulation (23). In the current review, we will first describe the role of epigenetics in T1D, before exposing the interactions between the gut microbiota and the epigenome. We will discuss the possible role of diet in modulating the gut microbiome, and current pre-clinical evidence that supports the approach of targeting the gut microbiome in management or treatment of T1D.

## Epigenetics in T1D

The symbiotic interplay between commensal bacterial communities and the host involves sophisticated molecular mechanisms of regulation at the epigenetic level. This involves the modification of gene expression as opposed to direct interference with the encoded DNA sequences. Metabolic pathways involved in gut microbiota crosstalk are essential in modifying the heritable changes in gene expression (28). These changes involve most notably patterns of DNA methylation, histone modification, and microRNA (miRNA) regulation (28–30). These different categories of epigenetic modification are found to be associated with insulin secretion and emerging conditions leading to T1D. Illustrations of this can be found in the studies discussed below.

Methylation of the DNA in the coding region of a target gene leads to CpG dinucleotides formation affecting the expression of this gene. Furthermore, hyper and hypo-methylation can be differentiated. While hypermethylation has shown to be responsible for gene silencing, hypomethylation has been shown to induce higher gene expression (23). Various studies have reported such an association between methylated genes and T1D (31–33). The best findings that gene methylation can condition T1D as an epigenetic factor are found in studies carried out in monozygotic twins. Raykan and colleagues conducted DNA methylation analysis of purified CD14+ monocytes from 15 T1D-discordant monozygotic twin pairs, and reported the hypermethylation of 54 genes and hypomethylation in 74 genes (34). In fact, the monocytes are immune effector cells that give rise to tissue macrophages that have been associated with the destruction of the islets cells and thus causing insulin deficiency (34). The authors identified 58 hypomethylation and 74 hypomethylation in variable positions and associated with T1D. Among the hypomethylated genes we can identify for example the hypomethylation of HLA class II gene, HLA-DQB1, which carries the highest single genetic risk for T1D (34). Similarly, another study conducted by Stefan and colleagues focused on DNA methylation profiles in B-cells from 3 monozygotic twins concordant and discordant for the disease (35). In this study, the authors identified 88 CpG sites in which 55 were hypermethylated and 33 were hypomethylated. This showed significant differences in DNA methylation between children with and without the disease; most significantly the hypermethylation of HLA-DOB and HLA-DQA2 genes (35). Another study conducted by Elbouwarej and colleagues provided strong evidence that hypomethylation of CpG sites within the promoter region in discordant monozygotic twins is related to T1D (36). Moreover, it has been shown that the decrease in the immune tolerance in T1D can be regulated by DNA methylation. A study conducted by Li and colleagues showed that the genomic DNA methylation in CD4+ T cells from the latent autoimmune diabetes in adults (LADA) with a subgroup of type 1 diabetes, was significantly increased compared to the controls (37). Also, the same study showed a decrease in the level of the hypermethylated FOXP3 promoter in CD4+ T cells of LADA patients compared with controls; a biomarker of T1D (37). On the other hand, many studies showed variation of DNA methylation within the insulin region (INS), which represent the second most important locus associated with T1D (38–42). In fact, the variation of the DNA methylation within the INS gene is suspected to regulate INS gene transcription in the pancreatic β-cells as well as the medullary thymic epithelial cells. These two tissues are known to be the sites where INS gene is expressed and to be the center of the mechanisms of T1D (43). One study highlighted the relationship between the immune response the DNA methylation changes at *Ins1* and *Ins2* genes that occurs in both β-cells of non-obese diabetic mice and human β-cells (41). This study suggests that increases in cytokine levels associated with T1D was associated with DNA methylation and decreased in insulin mRNA levels in β-cells (41).

Histone proteins allow the tight condensation of nuclear DNA into chromatin. Furthermore, their modifications cause an alteration in chromatin stability followed by abnormal DNA repair. Many enzymes are involved in epigenetic modulation of histones. These include: acetyltransferases (HATs) which add an acetyl group, histone deacetylases (HDAC) which removes them, histone methyltransferases (MHTs) which add methyl groups to lysine, and arginine residues in histone proteins that may enhance or diminish accessibility of DNA, and histone demethyltransferases (DMHTs) which removes them (44). Studies by Miao showed that various histone modifications are associated with T1D (45, 46). In these studies, a variety of different histone modification of genes (H3K9Ac, H4K16Ac, H3K4me3, H3K9me2,3, H3K27me3) were evident in T1D patients (45, 46). Monocytes from T1D patients had lower levels of H3K9Ac 4 kb upstream of HLA-DRB1 and higher levels of H3K9Ac 4 kb upstream of HLA-DQB1 (46). Similarly, a study conducted on the genomic DNA methylation in CD4+ T cells from LADA investigated whether the histone acetylation of CD4+ T may be involved in the development of LADA. The study showed that the expression of acetyltransferase CREBBP in LADA patients decreased, and that the expression of histone deacetylases HDAC1 and HDAC7 increased (47). Liu and colleagues concluded that a reduction in the histone H3 acetylation contributed to the pathogenesis of LADA (47).

Other epigenetic regulatory mechanisms make use of miRNAs that are involved in the posttranscriptional level of gene expression regulation through direct mRNA degradation or inhibition. A number of studies have revealed that miRNA can participate in the autoimmune damage of β-cells, regulation of insulin synthesis and secretion, and consequently in the pathogenesis of T1D (48, 49). Similarly, it has been shown that an increase in the level of miRNA-21, 29, 34a, and 146a was associated with β-cells dysfunction induced by proinflammatory cytokines (50, 51). Moreover, the study of the genome-wide miRNA expression profiles of T regulatory cells in T1D patients showed a significant increase of the level of miRNA-510 accompanied by a decrease in the level of miRNA-342 and miRNA-191 (52). Furthermore, various studies using cultured cells and animal models of T1D revealed that miRNAs can participate in the autoimmune damage of β-cells and the regulation of insulin secretion and synthesis (46). In fact, Ruan and colleagues showed that 2 genes of the NF-kB family activated the miR-21 gene promoter and thereby increased miR-21 RNA levels; this consequently decreased the level of programmed cell death protein 4 (PDCD4). The deficiency of PDCD4 in pancreatic β-cells renders them resistant to death (46). Thus, the NF-κB–miR-21–PDCD4 axis plays a crucial role in T1D and represents a unique therapeutic target for treating the disease.

## Effects of Microbial Metabolites on the Epigenome and T1D

**Figure 1:**
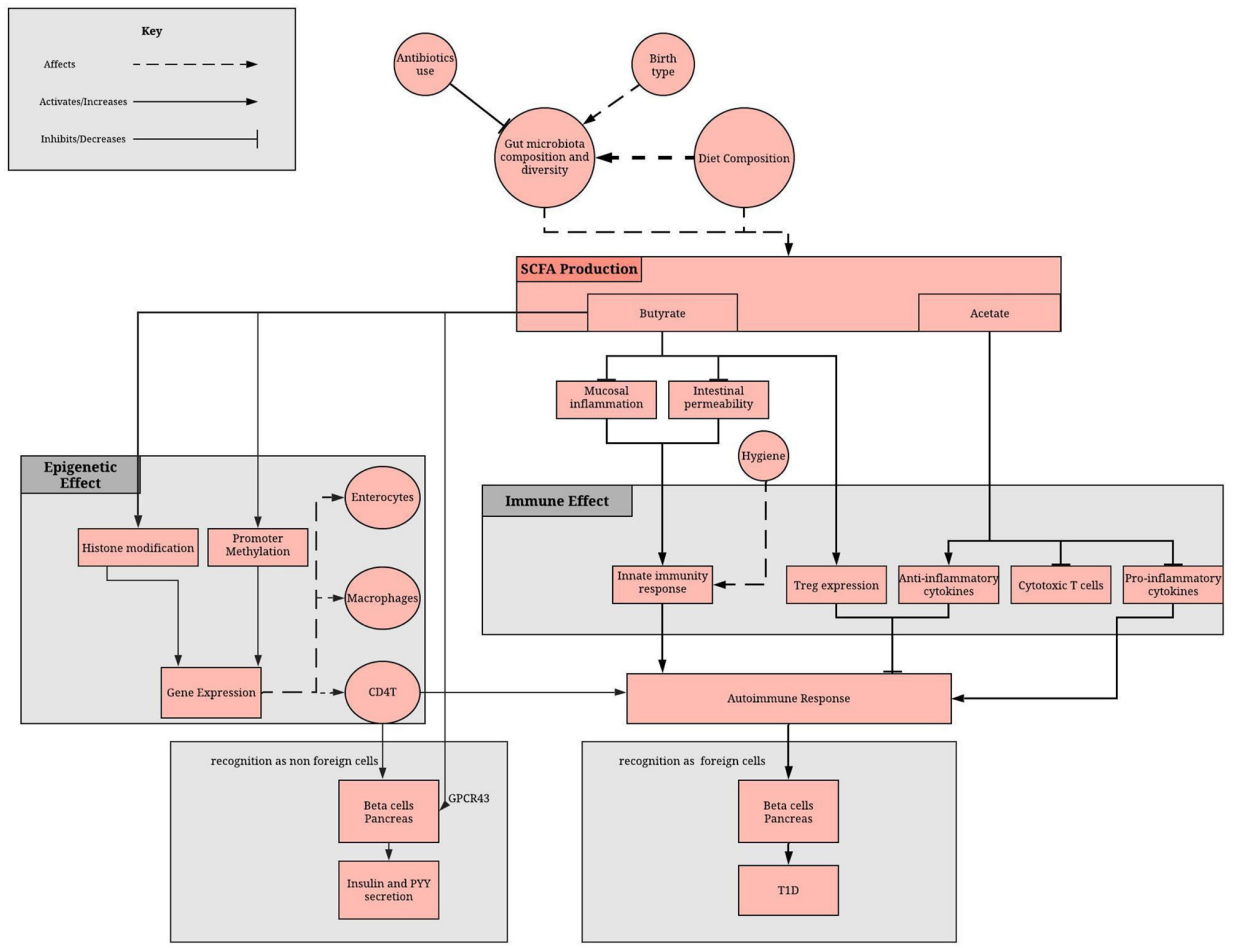
Role of the gut microbiome in epigenome and immune system regulation via the SCFA. SCFA production depends on gut microbiota composition and diet. SCFAs, mainly butyrate and acetate, reduces the inflammatory and increases the anti-inflammatory response of the adaptive immunity. Moreover, butyrate methylates promoter regions, affecting gene expression in enterocytes, macrophage and immune cells. A lack of SCFA can result in disruption of these processes, which can result in failure to recognize beta cells as non-foreign cells and therefore an autoimmune damage of pancreatic beta cells.

Recent evidence has suggested that the gut microbiota is an important mediator between diet and the establishment of the host epigenome and described how some of the secreted metabolites can affect DNA methylation and histone modifications in host for several diseases (53). However, the molecular mechanisms underlying this complex crosstalk, that is fundamental in achieving and maintaining homeostasis with the host, remain largely unknown (54–56). Butyrate, acetate, polyphenols and vitamins all play important roles in epigenetic regulation of T1D (Figure 1). They are either derivatives of gut microbes and/or are affected by gut dysbiosis.

**Butyrate** has shown to inhibit the activity of HDAC through its ability to suppress NF-κB activation, repressing the synthesis of interferon gamma and increasing the expression of peroxisome proliferator activated receptor-γ (57). A study conducted in 2015 showed that gut microbiota has an influence on the epigenome of T1D, where dysbiosis in T1D was accompanied by a decrease in butyrate production leading to the development of a permeable gut followed by development of autoimmunity for T1D (57). Another study supported this finding and provided evidence that butyrate has a protective effect on the development of anti-islet cell autoimmunity, associated with differences in the composition of mucin-degrading bacteria and the early introduction of complex food in a cohort of 44 children with and without persistent anti-islet cell autoantibodies (53). A study conducted by Khan and colleagues aimed to investigate the protective role of butyrate on islet β-cell function, proliferation, apoptosis, and glucose homeostasis in T1D mice and revealed that treatment with a salt of butyrate (sodium butyrate) enhanced insulin levels and glucose homeostasis, while reducing blood glucose through HADC inhibition and histone acetylation (58). HDAC inhibition has been associated with insulin gene regulation, β-cell proliferation, and differentiation, as well as the protection of beta cells from apoptosis, thereby counteracting the effects of diabetes. Prior to treatment with butyrate, it was found that almost all the beta islet cells in the diabetic mice were deprived of the endocrine cells, but results post treatment revealed that the cellular damage of islets had been ameliorated, highlighting the protective role of butyrate at both the molecular and tissue level (58). Further, the protective role of butyrate treatment was confirmed by evaluating the expression of transcription factors involved in glucose homeostasis, such as FOXO1. The aforementioned transcription factor plays an imperative role in the regulation of gluconeogenesis and glycogenolysis by switching on genes involved in the transcription of glucose-6-phosphatase, fructose-1,6-bisphosphatase and other enzymes involved in the said metabolic pathways (58). This indirectly increases hepatic glucose production. Further research illustrated that butyrate had significantly downregulated the expression of the FOXO1 transcription factor through mechanisms involving phosphorylation, facilitating insulin signaling and contributing to the improvement of glucose homeostasis.

**Acetate** is involved in maintaining homeostasis within the mammalian gut, wielding a potent role in the modulation of inflammatory cytokines by downregulating their expression but achieving the opposite effect for anti-inflammatory cytokines (59). An experiment carried out on NOD mice using the immunomodulatory peptide drug Glatiramer Acetate (GA), reported that it was effective at reducing the diabetic rate in the mice and ameliorating insulitis. This coincided with the increased expression of the transcription factor FOXP3, which is involved in regulating the proliferation and differentiation of Treg cells, and was revealed to have mediated the elevated production of IL-4 (60). Thus, the study connotes that GA and dietary acetate can be utilized as potential treatment for T1D through the induction of Tregs (CD4+CD25+ in particular) and that their enhanced function is partly due to the increased production of the anti-inflammatory cytokine IL-4.

**Polyphenols** are commonly found in fruits and vegetables, and are also produced as a product of the microbial metabolism of dietary foods in the GIT, where they are converted into various aromatic SCFAs such as phenylbutyrate. Additionally, polyphenols are known to inhibit HDAC activity, which may serve as a therapeutic benefit in neurodegenerative diseases such as Alzheimer's, Parkinson's, and Huntington's disease (61). Recent research has proposed that the metabolites exhibit anti- inflammatory properties and can therefore be used to avert chronic inflammation. Furthermore, they are believed to promote the secretion of immunoglobulins and maintain the function of the mucosal barrier of the gut (62). Despite the positive outcomes from extensive research, however, the exact mechanism of polyamine's actions is yet to be elucidated.

**Vitamins**, including folic acid, B2, B12, and B6, have been shown to be dietary- and microbiota-dependent, being susceptible to dietary intervention and gut dysbiosis (63). These vitamins play a fundamental role in generating S-Adenosylmethionine (SAM), the main methyl-donating substrate for DNMTs and HHMTs, the enzymes used to modulate the histones. In this context, it has been shown that many commensal gut bacteria such as *Lactobacillus* and *Bifidobacterium* species are very important in folic acid production, which confer them high consideration to be used as probiotics as we will show later (64).

Taken together, these data suggest that short chain fatty acids (SCFA), such as acetate and butyrate, which are products of the fermentation of non-digestible carbohydrates, such as dietary fibers and resistant starch, seems to possess predominantly beneficial effects including: the reduction of mucosal inflammation in the GIT, strengthening of the epithelial defense barrier to avoid pathogenic infections, and the prevention of insulin resistance and diabetes, among several others. Altered microbiota and SCFAs deficiency has been shown to be primary causal factors triggering T1D (65).

Consequently, a diet low in fat and gluten and high in resistant starch may be the primary driver of increasing the bacterial community responsible for producing butyrate (57). A diet rich of omega-3 polyunsaturated fatty acids (PUFA) induces a decrease in *Faecalibacterium*, often associated with an increase in the *Bacteroidetes* and butyrate-producing bacteria, as well as an increase in the production of anti-inflammatory compounds (66). The aforementioned studies provide a step in the direction of a functional understanding of the role played by the gut microbiome in the pathogenesis of T1D and indirectly the influence of the gut microbiota and the secreted metabolites on the epigenome of T1D. However, these finding needs to be validated by larger sample size cohorts and also include metagenomics data.

## Gut Microbiome and T1D

The residential community of the host gut microbiota is responsible for a variety of physiological functions associated with maintaining normal systemic and intestinal immunity, and also metabolic homeostasis (67). Gut microbiota diversity has been strongly implicated in the disease etiology of T1D and many studies have shown gut microbiota composition differences between healthy hosts and T1D hosts or people with heightened T1D risk (24).

Consistent with animal models, a number of clinical studies support that changes in gut microbiota diversity are strongly associated and might even be involved in etiology of human T1D (Table 1). It is responsible in particular for disease progression in infants, as well as patients who are at an especially high risk, usually due to genetic predisposition. A considerable number of children who progress to clinical T1D before puberty showed detectable levels of autoantibodies associated with T1D even before the age of 3 years (69).

**Table 1:** Pre-clinical and clinical studies on dysbiosis in T1D.

**Study**	**Study design**	**Main observations in gut microbiome**
Wirth et al. (68)	Rat model- control vs. streptozotocin -induced T1D.	T1D rats showed heightened levels of *Proteobacteria* and a reduction in normally dominant phyla (*Firmicutes* and *Bacteroidetes*).
Siljander et al. (69)	Case control study in 15 children with T1D, 15 children with maturity-onset diabetes pf the young 2 (MODY2) and 15 healthy children.	T1D was associated with a significantly lower microbiota diversity than controls, a significantly higher relative abundance of *Bacteroides, Ruminococcus, Veillonella, Blautia*, and *Streptococcus* genera, and a lower relative abundance of *Bifidobacterium, Roseburia, Faecalibacterium*, and *Lachnospira*. Proinflammatory cytokines, lipopolysaccharides (LPS). Gut permeability was significantly increased in T1D. T1D when compared with healthy control subjects.
Brugman et al. (70)	“In Bio-Breeding” diabetes-prone (BB-DP) rats.	Before the onset of T1D, there was a difference in the composition of gut microbiota between the rats that went on to develop T1D and those that did not.
Roesch et al. (71)	BB-DP rat model T1D vs. controls.	T1D had higher levels of *Bacteroides, Eubacterium*, and *Ruminococcus* accompanied by lower levels of *Lactobacillus, Bryantella, Bifidobacterium*, and *Turicibacter*.
Davis-Richardson et al. (72)	76 subjects with high genetic risk for T1D. Stool analysis from 4–6 month to 2 years old.	Results showed that 29 out of the 76 subjects, showed a specific increase in the relative abundance of *Bacteroides dorei*; associated to the onset of T1D in the Finnish children.
Murri et al. (73)	Case study that included 16 healthy children and 16 children with T1D.	Observed an abundance of *Actinobacteria* and *Firmicutes a*nd a lower ratio of *Firmicutes to Bacteroidetes* in the children with T1D than in the healthy children.
Giongo et al. (74)	Case study of four children using matched case controls.	Decrease in *Firmicutes* levels and an increase in the *Bacteroidetes* levels as children developed autoimmune disorders.
Vatanen et al. (26)	Analysis of metagenome in 783 children with T1D or predisposition or first degree relative with T1D.	Controls showed more genes that were related to fermentation and the biosynthesis of short-chain fatty acids (SCFAs) (26).

It has been proposed that the loss of higher diversity in patients with T1D when compared with healthy control subjects might be related to the autoimmune process (69, 75). The changes in gut microbiota composition early in development is useful in predicting the onset of T1D-related autoimmunity, particularly in infants who are genetically predisposed (69, 72, 75).

This might explain why some have proposed a role of the mode of delivery in T1D incidence, while in fact, it is probably a cofounding factor, where the observed increase abundance of *Bacterioides* in vaginally delivered infants, compared to those born by C section, is due to the infant's contact with the mother's vaginal microbiota upon passing through the birth canal (76–78).

The importance of gut microorganisms is manifest in the vital functions they perform; this includes intestinal epithelium protection, intestinal repair, prevention of the excessive abundance of certain strains of microorganisms and aggravated competition of others, and most notably, carbohydrate fermentation (79). The latter process is especially important in that it yields SCFAs as microbial metabolites which are implicated in a variety of regulatory functions, including modulation of the immune system (80). In fact, gut microbiota are important modulators of both systemic and intestinal immunity since they affect the migration and function of both leukocytes and lymphocytes. They facilitate the discrimination between host cells and pathogenic microbes, which is modulated by the expression of regulatory T cells. In this context, various studies identified *Bacteroides*, which is an acetate and propionate producing bacteria, as the main genus leading to T1D associated dysbiosis (69, 81, 82). In fact, *Bacteroides* are proposed to contribute to chronic inflammation by the impairment of the barrier function of the epithelial cell layer (82). Although, many of the *Bacteroides* can be the origin of the presence of significant higher level of LPS in T1D, which act as a molecular link between gut microbiota, inflammation, and T1D (6, 69), it is important to note that not all *Bacteroides* species induce the same level of endotoxemia.

Unbalanced gut microbiota was shown to affect also the integrity and permeability of the gut barrier (79). In experimental conditions, early onset autoimmune diabetes has shown to be accompanied by abnormalities of the gut barrier in the form of increased intestinal permeability. It is also accompanied by a decrease in mucus production, which constitutes another important barrier and controls the type of commensal bacteria residing in the epithelium (83–86). Taken together, the abundance, the diversity, the stability, and the connectivity of gut microbiota are associated with T1D development.

## Diet, Gut Microbiota, and T1D

Diet is one of the major factors that shape the composition of the intestinal microbiota (44). The diverse bacterial community colonizing different regions of the gastrointestinal tract has been associated with the exposure of the host to certain metabolites following the fermentation of dietary intakes (5). Many studies have shown that exposure to different types of diet at different stages of life may be connected to the development of preclinical and clinical T1D (87–89). As an example, it has been shown that maternal intake of fatty acids and their food sources during lactation may affect the preclinical and clinical type 1 diabetes development in the offspring (89).

The gut microbiota is involved in a number of physiological activities, in addition to its ability to influence lipid and glucose metabolism, and the mucosal immune response. Therefore, it contributes to both systemic immunity and inflammation in the gastro intestinal track (GIT). This is achieved through targeting the endocrine, immune and neuronal cells of the liver and GIT through the metabolites produced by the bacterial fermentation (90). A study conducted by Marino and colleagues involved implementation of a diet-based approach to induce dysbiosis in NOD mice. It was found that the mice developed T1D, which was preceded by the infiltration of the pancreatic islets of Langerhans by abnormal lymphocytes (insulitis). The study demonstrated that a diet high in the two SCFAs (butyrate and acetate) significantly inhibited the onset of the disease (65). The NOD mice were fed diets enriched with either acetylated or butylated high-amylose maize starch; these release significant amounts of their corresponding metabolites as a result of bacterial fermentation. The results indicated that both diets increased the levels of the two metabolites in both feces and blood, thereby protecting the mice from the manifestation of insulitis and T1D (65). Furthermore, the acetate rich diet is thought to predominantly inhibit the proliferation of effector T cells, compromising the activation of cytotoxic T cells and B lymphocytes, ultimately diminishing the effectiveness of the immune system (65, 91). Additionally, the mice exhibited a reduced availability of cells that were unable to perform their function as antigen presenting cells and showed an elevated level of *Bacteroides* amongst the gut microbiota, which contributes to protection against T1D. The aforementioned effects, however, were absent in the mice fed with a butyrate rich diet. Instead, the mice exhibited an expansion of regulatory T cells (Tregs) and an increase in the expression of occludin; a membrane protein that resides at tight junctions along the colon. Therefore, the diet significantly stabilized the integrity of the mucosal barrier (65). Furthermore, Sun and colleagues showed that the production of butyrate as fermented metabolite was associated with the expression of cathelicidin-related antimicrobial peptide (CRAMP) in the β-cells of NOD mice (92). Actually, this peptide presents a protective effect against T1D development (92). Furthermore, compared to the children who eventually developed T1D, the butyrate-producing bacteria were more abundant in the control group (82). It is therefore conceivable that the ratio of butyrate-producing to other SCFA-producing gut bacteria play a role in T1D.

Previous studies have indicated that proteins can alter the gut microbial composition and affect T1D development in humans and animals (93). A pilot study involving 230 infants at risk of T1D showed that supplementation with a diet rich in hydrolyzed casein (proteins accounting for up to 45% in human milk), was accompanied by a 50% reduction in the incidence of islet autoimmunity (94, 95). The protective effect of the hydrolyzed casein intake may be explained by the decrease in permeability of the epithelium cells by the peptides released after hydrolysis (96). Furthermore, it has been shown that a diet supplemented with gluten can lead to coeliac disease (CD) and may also influence the development of T1D (97, 98). In this respect, Cosnes and colleagues showed that a gluten-free diet plays a role in preventing the development of autoimmune disease (99). A study conducted on NOD mice showed that compared with a gluten-containing diet, a gluten-free diet can delay the onset and decrease the incidence of T1D along with increasing the number of T regulatory cells (100). In the same study, Marietta and colleagues found a decreased number of *Bifidobacterium, Tannerella*, and *Barnesiella* species, and an increased number of *Akkermansia* in the intestine in the presence of a gluten free diet, suggesting the protective role of this diet. Moreover, adding gluten to the gluten-free diet modulates the intestinal microbial composition by increasing the numbers of *Bifidobacterium, Tannerella*, and *Barnesiella* species and decreasing the number of *Akkermansia*. Thus, these data demonstrate that gluten-free diet plays a role in modulating the function of β-cells by changing the intestinal microbiota, which may influence the incidence of T1D.

While the macronutrients participate in shaping and modifying the gut microbiota, it is imperative that the effects of micronutrients receive attention for their contributions, particularly vitamin A, zinc, and iron. A deficiency in vitamin A alters the ratio of *Firmicutes to Bacteroidetes* and decreases the level of bacteria responsible for the synthesis of butyrate. This, in turn, causes hosts to become susceptible to the development of various chronic diseases, including diabetes (101). Additionally, the retinoic acid metabolite of vitamin A is capable of inhibiting the differentiation of pro-inflammatory Th17 cells derived from IL-6 (amongst the other necessary development cytokines). It also promotes the differentiation of anti-inflammatory Treg immune cells, which are involved in obstructing the manifestation of T1D. The defenses provided by a sufficient iron intake were found to increase the Enterobacteria and reduce the population of *Lactobacilli* in African children, both of which are involved in shaping the gut microbiome. This results in the secretion of less lactic acid in the GIT, thus promoting the proliferation of a greater diversity of bacterial species; consequently avoiding dysbiosis (6). Moreover, supplementation of zinc has been shown to increase bacterial diversity and activity in the ileum of piglets (102), but a deficiency of zinc can impede the inflammatory response, increasing the development of T1D (103). Finally, the intake and metabolism of the polyphenols has been shown to increase the abundance of bacterial species in the microbiome, such as *Bifidobacterium* and *Lactobacillus spp*., enhancing the production of SCFAs, again beneficiary in managing the development of T1D (104–106).

These findings illustrate that diabetes is a multisystemic disorder, and risk and can be reduced by ensuring an intake of a healthy balance of essential dietary nutrients.

## Future of T1D Management by Microbiome Therapy

### Modulation of T1D Susceptibility by Probiotics

According to the FDA and WHO, probiotics are defined as “live micro-organisms which can provide health benefits on the host when administered in adequate amounts” (107, 108). Traditionally, there are many different probiotics that are widely used: *Lactobacillus rhamnosus, Lactobacillus reuteri, bifidobacteria*, and certain strains of *Lactobacillus casei, Lactobacillus acidophilus*-group, *Bacillus coagulans, Escherichia coli* strain Nissle 1917, and certain enterococci, especially *Enterococcus faecium*SF68 (109). Probiotics may be added individually or in mixture of different strains that work in synergy and have additive benefits (109). The function of these probiotics varies within the same species, and may exhibit different benefits when used individually or in formulation. Furthermore, benefits may also differ according to patient group (106).

Since probiotics have been recognized for their beneficial effects on health, they have been used as potential dietary supplements (110). Probiotic health benefits are not only limited to the intestinal tract, but also include amelioration of many diseases and disorders including CVD, T2M, and T1M (111–114). The administration of certain strains of probiotics has resulted in several health benefits for T1D model animals as well as patients as outlined in Tables 2, 3. In fact, de Olveira and colleagues demonstrated that the consumption of probiotics decreases/eliminates dysbiosis and the breakdown of the gut epithelial barrier leading to the normal regulation of the gut membrane integrity and permeability (129). This effect was confirmed by a study of T1D in Bio-Breeding, Disease Prone (BB-DP) rats, where *Lactobacilus johnsonii* N6.2 was used as a probiotic (116). Several probiotic strains were also used to inhibit the proinflammatory and inflammatory cytokines as showed in different T1D animal models (116, 119, 122, 123, 125, 127). As an example, *Lactobacillus kefiranofaciens M and Lactobacillus kefiri* K used for STZ-induced C57BL/6 diabetic mice inhibits the proinflammatory and inflammatory cytokines TNFα and TH1 while increasing the production of anti-inflammatory cytokines IL-10 in pancreas (122). Also, the consumption of probiotic fermented milk, with *Lactobacillus rhamnosus* MTCC: 5957, *Lactobacillus rhamnosus* MTCC: 5897 and *Lactobacillus fermentum* MTCC, was associated with significant reduction in serum inflammatory cytokines like IL-6 and TNFα in STZ-induced diabetic rat Moreover, *Bifidobacterium* spp. was also responsible for reducing blood glucose levels significantly and increasing the protein expressions of insulin receptor β, which promotes the recovery of β-cells of pancreas, increasing insulin sensitivity in STZ-induced C57BL/6 diabetic mice. This is achieved by enhancing the function of the insulin signaling pathway. In summary, *Bifidobacterium* spp. can be considered as a promising bacteria for treating T1D (120). Since it has been known that SCFAs are able to ameliorate intestinal epithelial barrier integrity, which is responsible partly for protection against T1D (80), many scientists are interested to study the effect of some probiotics that modulate the gut microbiota leading to the increase of the production of this metabolite. The administration of *Lactobacillus* and *Enterococcus* probiotic strains from infant gut-origin increases short-chain fatty acid production via modulation of mice and human gut microbiome (130).

**Table 2:** Summary of major animal studies of probiotic interventions, their mechanism of action and their outcomes related to T1D.

**Name of Probiotic strains**	**Study type**	**Probiotic type and dose**	**Duration of intervention**	**Mechanism of action**	**Outcomes**	**Reference**	**Year**
VSL#3 (VSL Pharmaceuticals, Ft Lauderdale, FL, USA): 3 × 10^11^ CFU /g of **bifidobacteria** (*B. longum, B. infantis*, and *B. breve*), **lactobacilli** (*L. acidophilus, L. casei, L. delbrueckii* subsp. *L. bulgaricus*, and *L. plantarum*) and ***Streptococcus salivarius*** **subsp**. ***thermophilus***.	NOD mice	Three times a week of 3 mg/mouse, re-suspended in 100 μl PBS	28 weeks (from 4 to 32 weeks of age)	- Increases production of IL-10 from Peyer's patches and the spleen and with increase of IL-10 expression in the pancreas - Modulates GALT	- VSL#3 prevented autoimmune diabetes development in NOD mice - VSL#3 protected mice and induced immunomodulation by a reduction in insulitis severity	(115)	2005
*Lactobacillus johnsonii* N6.2	T1D in BB-DP Rats	1 × 10^8^ CFU per animal/day	21 day	- Changes in the native microbiota, host mucosal proteins, and host oxidative stress response - Decreases oxidative intestinal environment was evidenced by decreased expression of several oxidative response proteins in the intestinal mucosa (Gpx1, GR, Cat) - Decreases of the level of the pro-inflammatory cytokine IFNγ - Increases of the level of the tight junction protein claudin	- Delays or inhibits the onset of type 1 diabetes in BioBreeding diabetes prone rats.	(116)	2010
*Lactobacillus johnsonii* N6.2-Mediated	T1D in BB-DP Rats	1 × 10^8^ CFU/day through oral gavage	- BBDP rats received oral treatments daily until sacrifice at diabetes onset, or the culmination of the experiment at 140 day	- Diabetes resistance in LjN6.2-fed BBDP rodents was correlated to a Th17 cell bias within the mesenteric lymph nodes - Cytokines IL-6 and IL-23, respectively, were significantly higher within the mesenteric lymph nodes of LjN6.2-fed BBDP.	- Confirms resistance of T1D and the results published in (117)	(114)	2011
*Lactococcus lactis*	NOD mice	Not mentioned	Not mentioned	- Increases the frequencies of local Tregs accumulated in the pancreatic islets, - Suppresses immune response in an autoantigen-specific way - Preserves functional pancreatic islets and reduces in insulitis severity	- Reversal of autoimmune diabetes by restoration of antigen-specific tolerance: - Treatment strategy for 1TD in human	(117)	2012
*Lactobacillus plantarum* TN627	Alloxan-induced diabetes in rats	0.9 × 10^9^ CFU/ml/day	Not mentioned	- Improves the immunological parameters, - Protects the pancreatic tissues, and reduce the pancreatic and plasmatic α-amylase activities and level of plasma glucose in the treated as compared to the control group of rats - Reduces the pancreatic and plasmatic lipase activities and serum triglyceride and LDL-cholesterol rates - Increases the level of HDL-Cholesterol - Protects the liver and kidney functions by decreasing in serum aspartate transaminase, alanine transaminase, lactate dehydrogenase, and gamma-glutamyl transpeptidase activities, as well as creatinine and urea contents.	- Exhibits attractive *in vivo* antidiabetic effects that may be helpful in preventing diabetic complications in adult rats	(118)	2013
Bacterial LPS or Zymosan	NOD mice	25 μg/mouse/day in PBS	25 μg/mouse/day in PBS on days 1, 3, 5, 16, 18, and 20 after 12 weeks-old mice	- TLR2 and Dectin 1 engagement by zymosan promotes regulatory T-cell (Treg) responses against the pancreatic β-cell–specific antigen (Ag) - Zymosan induces a mixture of pro - and anti-inflammatory factors and Tregs, both *in vitro* and *in vivo* - Increased frequencies of IL-10–, IL-17–, IL-4–, and Foxp3-positive T cells, especially in the pancreatic lymph node	- Zymosan can be used as an immune regulatory adjuvant for modulating the T-cell response to pancreatic β-cell-Ag - Reverses early-stage hyperglycemia in T1D	(119)	2015
Bifidobacterium spp.	STZ-induced C57BL/6 diabetic mice	Not mentioned	5 weeks	- Reduces the blood glucose levels significantly - Increases the protein expressions of insulin receptor beta, insulin receptor substrate 1, protein kinase B (Akt/PKB), IKKα, and IκBα - Decreases both macrophage chemoattractant protein-1 (MCP-1) and interleukin-6 (IL-6) expression	- Promote the recovery of β-cells of pancreas or increase insulin sensitivity in mice by enhancing the function of the insulin signaling pathway may be the promising bacteria for treating diabetes	(120)	2015
*Lactobacillus reuteri*	STZ-induced C57BL/6 diabetic mice	10^9^ CFU / 0.3 ml directly into the stomach of mice 3 times per week	4 weeks	- Develops anti-inflammatory property by inhibiting osteoblast TNF-α signaling - Prevents TNF-α-mediated suppression of Wnt10b and osteoblast maturation markers	- Blocks the loss of bones open new avenues for use of probiotics to benefit the bone.	(121)	2015
*Lactobacillus kefiranofaciens* M and *Lactobacillus kefiri* K	STZ-induced C57BL/6 diabetic mice	1 × 10^8^ CFU per day	8 weeks	- Stimulates the secretion of GLP-1, inhibiting the proinflammatory and inflammatory cytokines, elevating the production of IL-10, and modifying the intestinal microbiota - Decreases of the level of the pro-inflammatory cytokine TNFa and TH1	- Potential ability of enhancing GLP-1 to mitigate the progression of type 1 diabetes *in vitro* and *in vivo*	(122)	2015
Oral Probiotic VSL#3	NOD mice	VSL#3 was administered through oral gavage three times a week	6 weeks (starting at 4 weeks until 20 weeks of age)	- Inhibits IL-1β expression while enhancing release of protolerogenic components of the inflammasome, such as indoleamine 2,3-dioxygenase (IDO) and IL-33 - Promotes differentiation of tolerogenic CD103^+^ DCs and reduces differentiation/expansion of Th1 and Th17 cells in the intestinal mucosa and at the sites of autoimmunity within the pancreatic lymph nodes (PLN) - Reduces the Teff/Treg cell balance in the gut mucosa and PLN	- Prevents autoimmune diabetes by modulating microbiota	(123)	2016
*Lactobacillus brevis* KLDS 1.0727 and KLDS 1.0373	STZ-induced C57BL/6 T1D mice	Not mentioned	4 weeks	- Capability of KLDS 1.0727 and KLDS 1.0373 strains as gad gene carriers to overexpress GABA significant effect on glucose level reduction in blood or insulin in plasma	- Inhibits the development of T1D in diabetic mice model	(124)	2018
Probiotic fermented milk with: Lactobacillus rhamnosus MTCC: 5957, Lactobacillus rhamnosus MTCC: 5897 isolated from dairy product (isolated from household curd) and Lactobacillus fermentum MTCC isolated from breast-fed human infant feces	STZ-induced diabetic rat	Not mentioned	6 weeks	- Decreases in the levels of blood glucose and HbA1c - Increases in the body weight, serum insulin, HDL-C levels in diabetic rats - Decreases in the inflammation, oxidative stress, and gluconeogenesis	- Can be useful for the complementary treatment strategies of diabetes and its associated complications	(125)	2018

**Table 3:** Summary of major human studies of probiotic interventions, their mechanism of action and their outcomes related to T1D.

**Name of Probiotic strains**	**Study type**	**Probiotic type and dose**	**Duration of intervention**	**Mechanism of action**	**Outcomes**	**Reference**	**Year**
*Lactobacillus johnsonii* N6.2	A double-blind, randomized clinical trial in 42 healthy individuals with no known risk factors for T1D	1 capsule/day containing 10^8^ colony-forming units of *L. johnsonii* N6.2 or placebo	8 weeks	- Significantly decreased the occurrence of abdominal pain, indigestion, and cephalic syndromes - Icreased serum tryptophan levels increased resulting in a decreased K:T ratio - Monocytes and natural killer cell numbers were increased significantly - Increases of circulating effector Th1 cells (CD45RO+CD183+CD196–) and cytotoxic CD8+ T cells	- Potential and safety therapeutic in prevention risk for T1D.	(126)	2017
*Lactobacillus rhamnosus GG* and *Bifidobacterium lactis Bb12*	96 children aged 8 to 17 years with newly diagnosed T1D	10^9^ UCF/day or placebo	6 months	- Modulate the immune system for preventing islet cell destruction - Improved the gut mucosal barrier	- Preserves cell function by reducing the risk of autoimmunity - Reduce/inhibits the growth of pathogens	(127)	2017
Probiotics	1039 individuals (mean age 46 ± 14 years, 45% men) with T1D and without end-stage renal disease	Not mentioned	2 years	- Decrease in obesity, body mass index - Regulated HDL-cholesterol, triglyceride components and blood pressure	- Better health related to diabetic disease	(128)	2017

In addition to the use of probiotics in animal models, the use of probiotics in humans at risk for T1D and in human T1D sufferers has also been investigated (Table 3). In fact, concerning those with elevated risk of T1D, studies revealed that an early supplementation of probiotics might lead to a decrease in the risk of islet β-cell autoimmunity (126, 131). Moreover, it has been shown that the use of probiotics in T1D adults was associated with a better glycemic control, reduced TLR4 inflammatory signaling, and also increased synthesis of glucagon-like peptide-1 (GLP-1), which is a hormone that stimulates the secretion of pancreatic beta cells leading to a decrease in blood sugar levels (132, 133). All these changes were associated with decreased incidences of T1D. A double-blind, randomized clinical trial in 42 healthy individuals with no known risk factors for T1D showed that the administration of 1 capsule/day containing 10^8^ UCF of *L. johnsonii* N6.2 or placebo for 8 weeks increased the circulating effector Th1 cells (CD45RO+CD183+CD196–) and cytotoxic CD8+ T cells. The data provided by this study, shows promise for the safety and feasibility of using *L. johnsonii N6*.2 in prevention trials involving subjects at risk for T1D (126). Another lactobacillus strain, *Lactobacillus rhamnosus*, in combination with another strain *Bifidobacterium lactis* Bb12, was administrated to 96 children aged 8 to 17 years with newly diagnosed T1D. A dose of 10^9^ UCF/day was given for 6 months. The mixture of these two strains can modulate the immune system thus preventing islet cell destruction and improving the gut mucosal barrier; this consequently reduces the risk of autoimmunity and reduces/inhibits the growth of pathogens (127). Furthermore, studies from 6 clinical centers in Europe and United States examined the association between supplemental probiotic use during the first year of life (0–27 days) and islet autoimmunity among children at increased genetic risk of T1D (131). Although, these TEDDY studies showed that early probiotic supplementation either by dietary supplements or though infant formula may reduce the risk of islet autoimmunity in children at the highest genetic risk of T1D, the result needs to be confirmed in further studies before any recommendation of probiotics use is made. Furthermore, it has been shown that the use of probiotics by T1D adult patients (mean age 46 ± 14 years, 45% men) is associated with better glycemic control and amelioration of metabolic syndrome, such as high triglyceride levels, high blood pressure (128). Altogether, these facts suggest (1) that early life exposure to probiotics can reduce the risk of T1D progression and (2) the potential of different probiotic strains in managing T1D via ameliorating the gut microbiota-immune system axis may have prompted the multiple, ongoing, new clinical human trials using probiotics to prevent/treat T1D. These studies are summarized in Supplementary Table 1.

### Modulation of T1D Susceptibility by Prebiotics

Like probiotics, some selective prebiotics have also been reported to be beneficial as shown in various animal and human studies (134–138). In 2017, Gibson and colleagues revisited the definition of prebiotics as “a substrate that is selectively utilized by host microorganisms conferring a health benefit” (139). In order to consider a food supplement as a prebiotic, it must have the following three criteria: (1) resistance to gastric acidity, hydrolysis by mammalian enzymes, and gastrointestinal absorption; (2) fermentation by intestinal microflora; and (3) selective stimulation of the growth and/or activity of intestinal bacteria associated with health and well-being (139, 140). Presently the most widely accepted prebiotics that fulfill the above criteria are lactulose, inulin, fructo-oligosaccharides (FOS), galacto-oligosaccharides (GOS) and the human milk oligosaccharides (HMO) (Table 4) (141–148). Despite the several updates concerning the concept of prebiotics over the years, it remains controversial whether a specific prebiotic must stimulate only one or various bacteria at the same time. Recent studies provided evidence that probiotics show some specificity for the prebiotics they may utilize (141–143). In fact, the ability to metabolize carbohydrates/prebiotics by intestinal bacteria was attributed to specific enzymes produced by these bacteria (Table 4). This further suggests that these bacteria present specific gene clusters in their genome that encode for enzymes involved in the metabolism of prebiotics; examples include β-Galactosidase from *Bifidobacterium adolescentis* (141), and β-fructofuranosidase by some Bacteroides (149). In human and animal models, the major fermented products of carbohydrate metabolism produced by gut microbiota are SCFAs. These bacterial metabolites appear to promote the development of a healthy immune system as shown by *in vivo* studies in animal models (Table 5) and humans (Table 6).

**Table 4:** Major prebiotics, their sources and the basis of bacterial specificity.

**Prebiotics**	**Sources**	**Specific related bacterial enzyme**	**Specific bacterial group**	**Fermented products**	**References**
Inulin type fructans Inulin Oligofructose		β-fructofuranosidase (fructanase)	Bifidobacteria Lactobacilli Bacteroides	Acetate, lactate Lactate Acetate, propionate	(146) (143) (149)
	Chicory (inulin)				
	Enzymatic hydrolysis of inulin Oligofructose				
Fructo-oligosaccharides (FOS)	Enzymatic synthesis from sucrose FOS	β-Galactosidase	Bifidobacterial	Acetate, lactate	(141, 142, 144, 145)

**Table 5:** Summary of major animal studies of prebiotic interventions, their mechanism of action and their outcomes related to T1D.

**Name of Probiotic strains**	**Study type**	**Prebiotic type and dose**	**Duration of intervention**	**Mechanism of action**	**Outcomes**	**Reference**	**Year**
Low antigen, hydrolyzed casein (HC)-based diet	LEW.1AR1-iddm ramodel	Fed daily	50 to 90 days	- Decreases in CD4+ Foxp3+ regulatory T (Treg) cells in pancreatic lymph nodes (PLN) - Decreases the expression of CD3+ T cells, CD163+ M2 macrophages and Foxp3+ Treg cells in the jejunum - decreases the Ifng/Il4 ratio, suggesting that the HC diet impacted M1/M2-associated cytokine balance - Corrects the gut immune cell deficits and increases the immunoregulatory capacity	- Prevention against T1D	(150)	2015
Dietary Resistant Starch	STZ-induced T1D Sprague-Dawley rats	550 g/kg diet	4 weeks	- Enhances GLP-1 and peptide YY secretion *in vivo*, which enhance β cell proliferation and insulin secretion improved the gut mucosal barrier - Protects the nephron	- Delayed the progression of diabetic nephropathy and maintained vitamin D balance in streptozotocin (STZ)-induced type 1 diabetic (T1D) rats develops normalized growth pattern in T1D	(151)	2016
Inulin-type fructans (ITFs), natural soluble dietary fibers with different degrees of fermentability from chicory root	NOD mice	Not mentioned	24weeks	- Increases CD25+Foxp3+CD4+ regulatory T cells, - Decreases IL17A+CD4+ Th17 cells, - Modulates cytokine production profile in the pancreas, spleen, and colon. - Increases the expression of barrier reinforcing tight junction proteins occludin and claudin-2, antimicrobial peptides β-defensin-1, and cathelicidin-related antimicrobial peptide - Enhances SCFAs production - Enhances *Firmicutes/Bacteroidetes* ratio to an antidiabetogenic balance and enriched modulatory *Ruminococcaceae* and *Lactobacilli*.	- Protects against autoimmune diabetes by modulating gut immunity, barrier function, and microbiota homeostasis. –> delays T1D development	(152)	2017
Wheat flour	NOD mice	Fed daily	72 days	- Lacks the epitopes linked with T1D - Reduces the levels of IFN-γ, a proinflammatory cytokine involved in the autoimmune pathogenesis of T1D - Increases the levels of IL-10, an anti-inflammatory cytokine potentially implicated in hindering T1D development	- Reduces the incidence of T1D.	(153)	2017
GABA therapy	STZ-induced C57BL/6 diabetic mice	Fed daily	11 weeks	- Increases Klotho levels in the serum, kidneys and pancreatic islets. - Stimulates the production and secretion of Klotho by human islet cells *in vitro*. - Stimulates the proliferation and insulin secretion by human islets - Klotho suppresses NF-κB activation - Increases insulin secretion	- Important implications for the treatment of T1D	(154)	2017
Caffeic acid-rich fraction from *Prunella vulgaris* L plant	Alloxan-induced diabetic mice	Fed daily	8 weeks	- Reduces blood glucose levels and improved *in-vivo* oxidative-stress - Inhibits the carbohydrate-hydrolyzing enzymes (alpha-amylase and alpha-glucosidase) - Reduces HbA1c levels - Increases serum-insulin levels	- Possess antidiabetogenic and antinociceptive properties could be a potential therapeutic agent to ameliorate T1D and related complications.	(155)	2018
Human milk oligosaccharides consisting of both long-chain, as well as short-chain structures	NOD mice	Fed daily	6 weeks	- Increases anti-inflammatory microbiota-generating metabolite [i.e., short chain fatty acids (SCFAs)] - Induces anti-diabetogenic cytokine profiles - Induces the development of tolerogenic dendritic cells (tDCs), priming of functional regulatory T cells, which support the protective effects - Increases butyrate production promoting mucin synthesis improving the intestinal barrier integrity	- Delays and suppresses T1D development in non-obese diabetic mice and reduces the development of severe pancreatic insulitis in later life - Vital in the protection of children at risk for T1D, supporting immune and gut microbiota development in early life.	(156)	2018

**Table 6:** Summary of major human studies of prebiotic interventions, their mechanism of action and their outcomes related to T1D.

**Name of Probiotic strains**	**Study type**	**Prebiotic type and dose**	**Duration of intervention**	**Mechanism of action**	**Outcomes**	**Reference**	**Year**
Dietary fiber	106 outpatients with type 1 diabetes; age 40 ± 11 years	fiber intake > 20 g/day	-	- Develop anti-inflammatory properties - Shows inverse association between some nutritional habits and highly sensitive -C-reactive protein (hs-CRP)	- Suggests that an increased consumption of dietary fiber > 30 g/day may play a role in reducing inflammation in individuals with type 1 diabetes. - Lower the risk of coronary disease	(157)	2014
Oligofructose enriched inulin	Randomized, double-blind, placebo-controlled trial in children aged 8 to 17 years with T1D for at least 1 year	Placebo (maltodextrin 3.3 g orally/day) or prebiotics (oligofructose-enriched inulin 8 g orally/day; Synergy1, Beneo, Mannheim, Germany)	12 weeks	- Changes gut microbiota, gut permeability and inflammation. - Decreases endotoxemia and reduces insulin resistance - Improves glycemic control	- Potentially novel, inexpensive, low-risk treatment addition for T1D	(134)	2016
Adjunct therapy with dapagliflozin	33 youths (14 males, median age 16 years, diabetes duration 8 years)	10 mg DAPA or placebo during dosing visits	-	- Reduces the insulin dose required as a medication - Improves the glycemic control by targeting the glucose level - Increases the urine glucose excretion	- Potential therapy in the pediatric age group by lowering insulin dose and increasing glucose excretion.	(135)	2017
Dietary fiber intake	111 outpatients with T1D	daily intake (<14 g fiber/1,000 kcal) or (≥14 g/1,000 kcal)	dietary intake was evaluated by 3-day weighed-diet records	- Reduction in the use of medicine for diabetes (insulin) and hypertension (ACE inhibitors) treatment - Shows lower the blood pressure - Shows lower body mass index (BMI) - No significant association between increased fiber intake (≥14 g/1,000 kcal/day) and serum levels of total cholesterol, HDL cholesterol, or LDL cholesterol.	- Associated with lower blood pressure levels in patients with type 1 diabetes	(136)	2018

In the last 5 years an increasing number of studies using animal models have shown that the intake of dietary fibers plays an important role in maintaining gut microbiota homeostasis; this leads to a delay in the development of T1D (Table 5). For example, feeding LEW.1AR1-iddm rats with a low antigen, hydrolyzed casein-based diet was shown to correct gut immune cell deficits and increase immunoregulatory capacity leading to protection against T1D (150). Similarly, feeding NOD mice with wheat flour for 72 days shows the same effect with a decrease in the levels of IFN-γ (a proinflammatory cytokine involved in the autoimmune pathogenesis of T1D) and an increase in the levels of IL-10 (an anti-inflammatory cytokine potentially implicated in hindering T1D development) (127). One study involved the feeding of NOD mice with chicory root, which is rich in Inulin-type fructans ITFs; a natural soluble dietary fiber with different degrees of fermentability. This 24-week treatment was protective against autoimmune diabetes due to its action of modulating gut immunity, improving barrier function, and promoting microbiota homeostasis. These factors resulted in the delay of T1D development (152). Furthermore, the intake of this prebiotic was found to enhance SCFAs which modulate the production of cytokines in the pancreas, spleen, and colon. Also, the intake of ITFs enhances the *Firmicutes/Bacteroidetes* ratio to an antidiabetogenic balance and enriches modulatory *Ruminococcaceae* and *Lactobacilli*. Altogether, these changes consequently increase the expression of the gut barrier by reinforcing tight junction proteins thus decreasing its permeability. Feeding NOD mice with Human milk oligosaccharides (HMOS), consisting of both long-chain, as well as short-chain structures, has also been found to increase the anti-inflammatory microbiota-generating metabolite SCFAs (156). In fact, the increase of butyrate production has been shown to promote mucin synthesis, thus improving intestinal barrier integrity. This has been found to delay and suppress T1D development in NOD mice, and to reduce the development of severe pancreatic insulitis in later life. This suggests that the supplementation of HMOS may be vital in the protection of children at risk for T1D by supporting their immune system and their gut microbiota development in early life. Supplementation of dietary resistant starch (the caffeic acid-rich fraction from Prunella vulgaris L plant) and the use of GABA therapy proved that those prebiotics had beneficial effects on animals and that they could form a potential therapeutic agents to ameliorate T1D and related complications (151, 154, 155).

The use of prebiotics in human subjects with T1D has also been investigated (Table 6). In a single-center, randomized, double-blind, placebo-controlled trial in children aged 8 to 17 years with T1D, the consumption of oligofructose and-enriched inulin for at least 1 year was shown to positively modulate intestinal permeability and reduce of inflammation by increasing the number of *Bacteroidetes* and lactic producing bacteria, thus improving glycemic control and reducing the chances of T1D occurrence (134, 138). Another study of 106 outpatients with type 1 diabetes and having an age of 40 ± 11 years, involved supplementation with dietary fibers; outcomes included a lower risk of coronary disease (157). Furthermore, this study suggested that an increased consumption of dietary fiber (>30 g/day) may play a role in reducing inflammation in individuals with T1D. Another study published in 2018, involved a group of 111 outpatients with T1D. Following a high fiber, prebiotic, dietary supplement, there was a subsequent reduction in the use of medicine for diabetes (insulin) and hypertension treatment (ACE inhibitors), and also a lowering of blood pressure (136). Moreover, in a randomized, double-blind, placebo-controlled, pilot study of children aged 8 to 17 years with T1D, the intake of oligofructose enriched inulin was accompanied by: (1) altered gut microbiota, gut permeability, and inflammation, (2) decreased endotoxemia and reduction of insulin resistance, and (3) improved glycemic control (134). This prebiotic may be a potentially novel, inexpensive, low-risk treatment addition for T1D. The positive effects of the intake of oligofructose can be explained as follows. This prebiotic is metabolized by different bacteria species like *Bifidobacteria, Lactobacilli*, or *Bacteroides* under the presence of the β-fructofuranosidase enzyme. It follows that they will produce different metabolites (acetate, lactate, and propionate) (140, 143, 149). Although the produced SCFAs has been shown to alter the host immune system and ameliorate T1D (104), a recent randomized controlled trial showed that oral butyrate administration for 1 month to 30 individuals with longstanding T1D did not result in any significant changes in innate immunity and islet autoimmunity (158).

Altogether, these facts demonstrate the potential of different prebiotics in preventing and managing T1D. As in the case with probiotics, their promise may explain the new, multiple, ongoing clinical human trials using prebiotics to prevent/treat T1D, which are summarized in Supplementary Table 2.

### Modulation of T1D Susceptibility by Symbiosis

Previous studies have shown that probiotics and prebiotics may improve glucose metabolism. In order to improve their potential therapeutic efficacy, they may be used together as “symbiotics.” This means that probiotics and prebiotics can complement each other or even work in synergy to provide a combined effect. There are several recent studies that have shown positive synergetic effects of symbiotics using animal studies and clinical human studies for different diseases including T1D (159–166). For example, the supplementation of a combination of *Bifidobacterieum animalis* ssp. *Lactis* (B420) as a probiotic and polydextrose as a prebiotic has been investigated using Ketogenic diet-induced C57Bl/6J diabetic mice. After this 4-week supplement regime, the mice showed an increased concentration of portal GLP-1, a decreased glycemic response, and lower plasma glucose concentration (Supplementary Table 3) (159). This improved the efficacy of metformin, a benefit for combining probiotics and/or prebiotics with antidiabetic drugs, which show promise for transferring this study to the clinical level.

## Limitations and Lessons Learned From Experimental Studies

Fundamental and pre-clinical studies support the promising role of probiotics in the management and treatment of diabetes. An important study by Cheng and colleagues showed that a short-duration diet that mimics fasting can induce regeneration of β-cells and rescue mice from not only T2D but also T1D (167).

Although both animal and human studies showed the promising effect of diet and pro/prebiotics in managing T1D, we should bear in mind that autoimmune diabetes observed in mice is not identical to those observed in humans (168). In fact, a major difference between these two species is the time of the T1D development. While NOD mice typically show the disease close to their maturity, human seroconversions peak has been shown to occur during the first 3 years of life (168). Moreover, what makes the comparison even more complicated is that nearly all autoimmune diabetic cases occur in adult NOD mice, whereas approximately half of the cases appear before adult age in humans (168).

Also, we should emphasize that most of the available clinical trials come from studies that have used different methodologies, which makes comparison difficult. Although the obtained outcomes hinder a clear conclusion, it reveals a clear trend. For example, the trials differ greatly in sample size; furthermore, homogeneity of the patients in terms of demographics, symptoms, and dietary habits is lacking. There are also inconsistencies in strains, dose, and duration of probiotic/prebiotic intervention that limit the possibility of identifying which probiotic/species/strain or therapy has contributed the most to prevent/delay the disease in humans with high risk and/or help in managing the disease in T1D patients. Moreover, human studies mainly reveal associations between diet, gut microbiota and T1D rather than causal relationship.

Furthermore, attention should be given to the safety of probiotics in clinical practice. In fact, although probiotics have an excellent overall safety record, they should be used with caution in susceptible individuals, particularly neonates born prematurely or with immune deficiency. This is because excessive immune stimulation may be caused by probiotics in vulnerable individuals (169). Moreover, some secondary effects have been presented in the presence of some probiotic strains. Fungal sepsis caused by *S. boulardii* has also been reported (170), along with minor bacteremia, sepsis, and cholangitis induced by *Bacillus subtilis* and by *lactobacilli L. rhamnosus* GG or *L. casei* (169–171).

An important feature of TD1 in humans is that by the time of diagnosis, it is difficult to revert it, and the influence of the diet and/or microbiome therapy might be dependent on the relative time-to-diagnosis. Some have suggested that microbiome therapy could constitute a preventive measure before seropositivity; others suggested the possibility of reversing T1D through isolated case reports. Indeed, a recent study showed that two cases of T1D in pediatric subjects were treated with supplementation of high dose vitamin D and omega 3 and a similar pattern of remission of the disease was observed, resulting in restoration and subsequent persistence of optimal metabolic control, one and 2 years after T1D onset (172). The study also showed that a minimal basal insulin administration (0.1 IU/kg/die) in a single evening injection was required. Although this study showed promise for this diet, larger controlled studies are needed to determine the effect of the proposed intervention to slow down or halt the progression of autoimmunity. Furthermore, although patients with T1D showed a reduced microbial diversity and a proinflammatory intestinal dysbiosis, studies revealed that microbiome therapy may contribute to the reduction of intestinal permeability, the reduction of inflammation and to the improvement of glycemic control in T1D patients. Moreover, we should bear in mind that during the first 3 years of life the gut microbiome undergoes dynamic changes before becoming more stable, which provides a time window between birth and 3 years of age to apply a diet and a supplementation of pre/probiotics to children with predisposition of T1D or first-degree relative(s) with T1D. In fact, during that period, guidance in developing an equilibrate gut microbiome, and training for the developing immune system are required in order to establish self-tolerance and to control inflammatory responses.

## Conclusions and Future Plans

**Figure 2:**
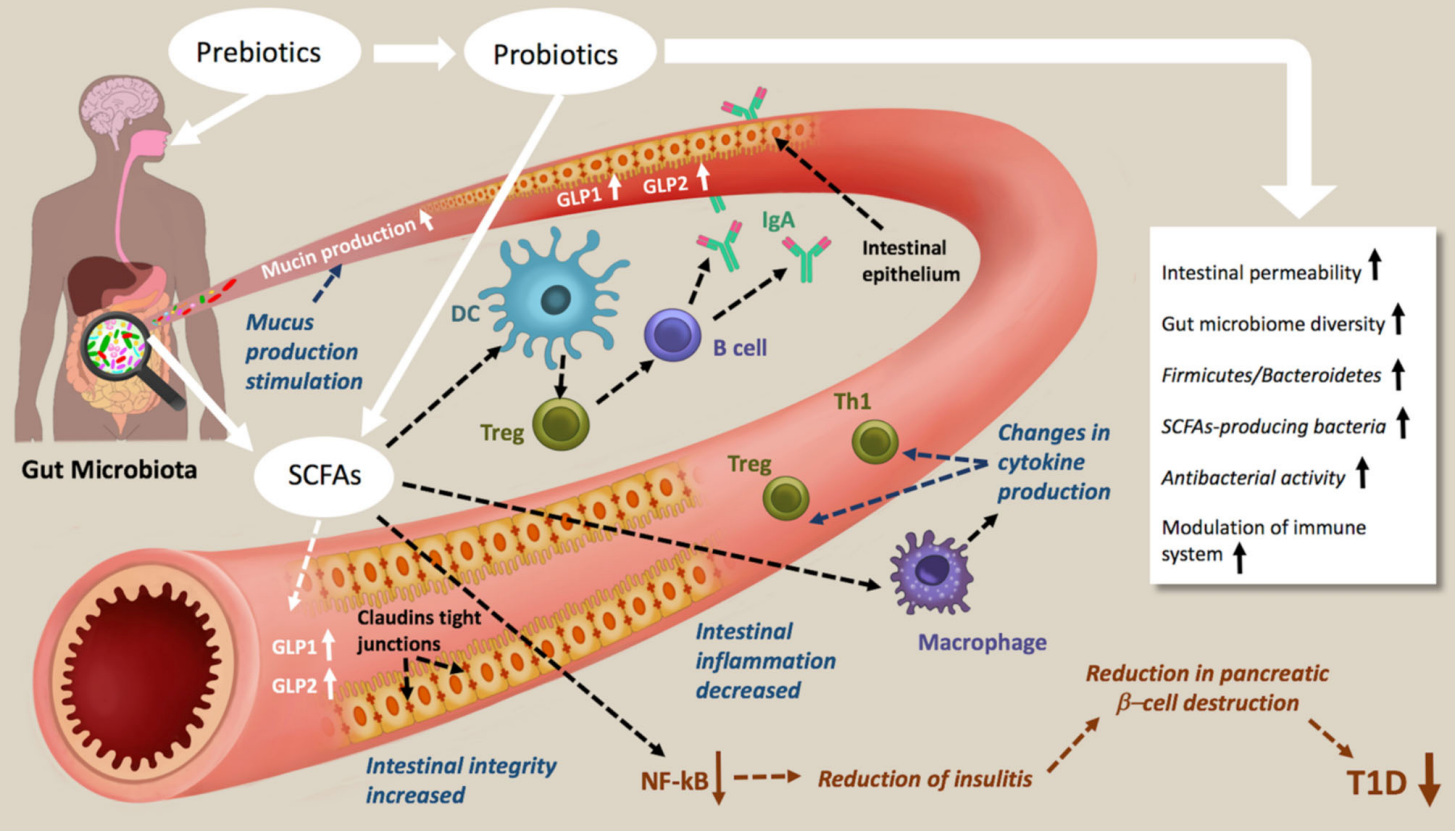
Schematic representations of prebiotic and probiotic actions in type 1 diabetes. Prebiotic and probiotic consumption increases the Gut microbiome diversity the *Firmicutes/Bacteroidetes*, the antibacterial activity, decreases the intestinal permeability and modulate the immune system. As results of increasing SCFAs production, mucin production, intestinal integrity and tight junction are increased, intestinal inflammation decreased. The NF-kB activation pathways are blocked, which lead to a reduction of insulitis, reduction in pancreatic β-cell destruction and therefore amelioration of T1D.

The change in expression of genes is non-genetically determined and depends largely on environmental factors. Recent advances in understanding the role of the gut microbiome in building immunity and its involvement in pathogenesis suggests an important approach to managing and treating T1D. Diet and nutrition play an important role in the modulation of the gut microbiome, and it is now clear that dietary fibers and the introduction of pre and probiotics could constitute powerful tools to correct the dysbiosis found in T1D patients. Metabolites produced by beneficial microbes, such as the SCFA can affect the epigenome, along with the differentiation and functioning of various cell types, including enterocytes, immune cells and pancreatic cells (Figure 2). Despite the available evidence on the impact of dietary gut microbial metabolites on the epigenome of T1D and the existing of significant association between diet, gut microbiota and epigenetics of host cells, a better understanding of epigenetic mechanisms is still necessary for the identification of the epigenetic pathways involved in T1D. Finally, the modulation of the gut microbiome for treating T1D shows great promise; this explains the launch of clinical trials aiming at repairing dysbiosis, either by dietary intervention or fecal transplants (173, 174). Although, the results of these trials might play an important role in our understanding of the environmental factors involved in the pathogenesis of T1D (27, 175, 176), establishing and validating functional regimens, like probiotics and prebiotics, in long term studies is necessary.

Furthermore, future studies should be better coordinated and well-characterized, with high quality and long-term sampling and data collection starting from pregnancy to the onset of T1D in high risk children. This may reveal the mechanisms by which diet and/or administration or pre/probiotics prevent and protect against T1D. Furthermore, we advocate for standardized and reproducible methods for DNA and RNA studies, followed by new approaches such as metagenomics, metaproteomic, and metabolomics applied to a large cohort of patients as well as intervention studies. This may, in the future, elevate our understanding of this topic and move this area of research from a description of effect, sometimes indirect, to translational applications.

## Author Contributions

AC conceived the study, critically supervised the project, revised the text, wrote some parts of the review and prepared the tables and the figure. CM helped in editing the text and sketched the figure. GB helped in editing the text and gave support in the literature review and writing. AA, TA and AA-S were responsible for scientific writing of the first two parts of the draft manuscript. All authors contributed to the proofreading of the manuscript and approved the final version of the manuscript.

## Conflict of Interest

The authors declare that the research was conductedin the absence of any commercial or financial relationships that could be construed as a potential conflict of interest.
