# Supplementary material for: Epigenetic Effects of Gut Metabolites: Exploring the Path of Dietary Prevention of Type 1 Diabetes
Source: Front Nutr. 2020 Sep 24;7:563605. doi: 10.3389/fnut.2020.563605 (PMC7541812; doi:10.3389/fnut.2020.563605)
Supplement: Supplementary file 1 [file Data_Sheet_1.PDF]

Epigenetic effects of gut metabolites: exploring the path of dietary prevention of type 1 diabetes

Ahmad Al Theyab[1], Turki Almutairi[1], Abdulla M Al-Suwaidi [1], Ghizlane Bendriss[1], Clare McVeigh[1], and Ali Chaari[1*]

[1] Premedical division, Weill Cornell Medicine Qatar, Education City, P.O. Box 24144, Doha, Qatar.

*** Correspondence:**

Corresponding Author

alc2033@qatar-med.cornell.edu

Table S1: Summary of ongoing human studies of probiotic interventions to T1D

NCT No.	Trial name	Interventions	Intervention Model	Outcomes	Estimated enrollment	Sponsors	Status
NCT03423589	Modulation of Type 1 Diabetes Susceptibility Through the Use of Probiotics	VSL#3	Single Group Assignment	- Alterations in Plasma-Induced Transcriptional Analysis<br>- Intestinal Microbiota	30	Medical College of Wisconsin<br><br>Milwaukee, Wisconsin, United States	Completed
NCT03032354	Probiotics in Newly Recognized Type 1 Diabetes	Drug: Lactobacillus rhamnosus GG and Bifidobacterium lactis BB12<br><br>Other: Placebo, (Placebo group)	Parallel Assignment	Insulin analysis<br><br>HbA1c analysis	96	-	Unknown
NCT03880760	The Effect of Probiotics on Type 1 Diabetes Mellitus in Children	L. johnsonii MH-68, B. animalis subsp. lactis CP-9 and L. salivarius AP-32<br><br>Other: Placebo	Parallel Assignment	Change in percentage of HbA1c<br><br>Change in concentration of blood glucose (AC)<br><br>Change in concentration of MIP-1β	80	China Medical University Hospital<br><br>Taichung, Taiwan	Recruiting
NCT03556631	Effect of Live Combined Bifidobacterium and Lactobacillus on Glycemic Control and Other	Drug: live combined Bifidobacterium and Lactobacillus Tablets	Parallel Assignment	hemoglobin A1c in percentage<br><br>weight in kilograms	30	-	Not yet recruiting

	Outcomes in Type 1 Diabetes						
NCT04141761	Probiotics in Newly Diagnosed T1D	Dietary Supplement: Visbiome<br><br>Other: Placebo	Parallel Assignment	Effect of Multistrain Probiotic on Immune System Inflammation as measured by plasma transcription analysis C-peptide decline<br><br>Effect of Multistrain Probiotic on broader Immune System Effects as measured by plasma-induced transcriptional analyses	60	Medical College of Wisconsin<br><br>Milwaukee, Wisconsin, United States	Recruiting
NCT03961347	Lactobacillus Johnsonii Supplementation in Adults With T1D	Drug: L. johnsonii Probiotic<br><br>Drug: Placebo Capsule	Parallel Assignment	Number of participants with at least one adverse event	62	UF Clinical Research Center<br><br>Gainesville, Florida, United States	Recruiting
NCT03961854	Lactobacillus Johnsonii in Children and Adolescents With T1D	Drug: L. johnsonii Probiotic<br><br>Drug: Placebo Capsule	Parallel Assignment	Number of participants with at least one adverse event	62	UF Clinical Research Center<br><br>Gainesville, Florida, United States	Recruiting
NCT02605148	TEFA Family Prevention: Glutenfree Diet to Preserve Beta-cell Function	Dietary Supplement: Gluten free diet<br><br>Dietary Supplement: Omega 3 fatty acid<br><br>Dietary Supplement: Vitamin D	Parallel Assignment	Change in first phase insulin response (FPIR) from IvGTT<br><br>Change in area under the curve (AUC) C-peptide<br><br>Change in glucose metabolism	60	Lund University, Department of Clinical Sciences Malmö<br><br>Malmö, Sweden	Enrolling by invitation

		Dietary Supplement: Probiotics		Incidence of Treatment-Emergent Adverse Events			
NCT04014660	Prevention av Autoimmunitet Med Laktobaciller	Dietary Supplement: Probiotic<br><br>Dietary Supplement: Placebo	Parallel Assignment	Autoimmunity	200	Clinical Research Center (CRC), Bldng 60:11 Malmö, Sweden	Recruiting
NCT02349360	Safety, Tolerability and Host Response to Lactobacillus Johnsonii	Biological: L. johnsonii N6.2<br><br>Biological: Placebo	Parallel Assignment	Number of Participants Reporting Adverse Events<br><br>Composite Measure of Blood Chemistry Profiles	42	University of Florida, Food Science and Human Nutrition Dept<br><br>Gainesville, Florida, United States	Completed
NCT04191525	Phase II Clinical Trial to Evaluate the Efficacy and Safety of the Treatment With BPL-1 in Adult Patients With Type 2 Diabetes Mellitus	Dietary Supplement: BPL-1 Probiotic capsules<br><br>Dietary Supplement: Placebo	Parallel Assignment	Fasting glucose<br><br>Glycated haemoglobin<br><br>Insulin	90	Hospital Universitario de La princesa<br><br>Madrid, Spain	Completed
NCT02861261	A Study on the Efficacy and Gut Microbiota of Berberine and Probiotics in Patients With Newly Diagnosed Type 2 Diabetes	Drug: 1. Berberine hydrochloride tablets ; 2. ProMetS probiotics powder<br><br>Drug: 1. Berberine placebo tablets ; 2.	Parallel Assignment	HbA1c<br><br>Gut microbiome<br><br>Fasting glucose levels	400	Ruijin hospital,Shanghai Jiao Tong University School of Medicine<br><br>Shanghai, Shanghai, China	Active, not recruiting

		ProMetS probiotics powder<br><br>Drug: 1. Berberine hydrochloride tablets; 2. Probiotics placebo powder<br><br>Drug: 1. Berberine placebo tablets; 2. Probiotics placebo powder					
NCT02728414	Probiotics Effect on Glucose and Lipid Metabolism and Gut Microbiota in Patients With Type 2 Diabetes	Dietary Supplement: probiotics<br><br>Dietary Supplement: placebo	Parallel Assignment	Change from baseline in fasting blood-glucose at 1 month<br><br>Change from baseline in fasting blood-glucose at 3 months<br><br>Cchange from baseline in glycosylated hemoglobin change at 1 month	100	Shanghai 10th People's Hospital<br><br>Shanghai, Shanghai, China	Unknown
NCT01250106	Probiotics as a Novel Approach to Modulate Gut Hormone Secretion and Risk Factors of Type 2 Diabetes and Complications	Dietary Supplement: Lactobacillus reuteri	Parallel Assignment	Insulin resistance<br><br>Gut hormones (GLP-1, GLP-2, GIP)<br><br>Insulin secretion	20	German Diabetes Center<br><br>Düsseldorf, Duesseldorf, Germany	Unknown
NCT03239366	A Study to Evaluate the Effect of BioK+ 50B® on Glycemic Control in a Type 2 Diabetes Population	Other: BioK+ 100% probiotic<br><br>Other: Placebo	Parallel Assignment	Change in HbA1c levels from baseline<br><br>Evaluation of the effects of Bio-K+50B® as compared with placebo after 12 weeks	130	Montreal Heart Institute<br><br>Montréal, Quebec, Canada	Recruiting

				of Treatment on different biochemical markers:<br><br>Evaluation of the intestinal colonisation effects with the 2 capsules of Bio-K+50B® (dosage of 100 billions bacterias) as compared with placebo<br><br>Evaluation of the safety profile of 2 capsules of Bio-K+50B® (dosage of 100 billions bacterias)			

Table S2: Summary of ongoing human studies of prebiotic interventions to T1D

NCT No.	Trial name	Interventions	Intervention Model	Outcomes	Estimated enrollment	Sponsors	Status
NCT02442544	Prebiotic Fiber Supplement in T1DM Children	Dietary Supplement: Prebiotic<br><br>Dietary Supplement: Placebo	Randomized	Glycemic control (serum hemoglobin A1c). Gut microbiota composition (mRNA in stool). Glycemic control (as measured by serum hemoglobin A1c)	38	Alberta Children's Hospital<br><br>Calgary, Alberta, Canada	Active, non-recruiting
NCT04114357	Effect of Prebiotics on the Gut Microbiome Profile and Beta Cell Function	Drug: Acetylated and Butyrylated High Amylose Maize Starch	Randomized	Change in the gut microbiome profile<br><br>Changes in the Short Chain Fatty Acid Levels in the gut.<br><br>Changes in Glycemia.<br><br>Changes in Beta cell Health	12	-	Not yet recruiting
NCT02903615	Optimising Health in Type 1 Diabetes	Other: Novel diet	Randomized	Glucose control<br><br>Inflammation<br><br>Gut microbiome	20	professor Katherine Samaras<br><br>Sydney, New South Wales, Australia<br><br>Garvan Institute to Medical Research<br><br>Sydney, Australia	Unknwon

NCT02569684	Effects of Prebiotics on GLP-1 in Type 2 Diabetes	Dietary Supplement: Prebiotic fibers: oligofructose and inulin<br><br>Dietary Supplement: Maltodextrin	Randomized	Changes in plasma GLP-1 in response to standardized test meal before and after six weeks of ingestion of prebiotic fibers or placebo	37	Oslo University Hospital<br><br>Oslo, Norway	Not yet recruiting

Table S3: Summary of major studies of symbiotic interventions, their mechanism of action and their outcomes related to T1D

Name of Probiotic strains	Study type	Prebiotic type and dose	Duration of intervention	Mechanism of action	Outcomes	Reference	year
Probiotic: *Bifidobacterieum animalis ssp. Lactis* (B420) and prebiotic; polydextrose	Ketogenic diet-induced C57Bl/6J diabetic mice	B420: 109 CFU/day, and polydextrose: 0.25 g/day	4 weeks	- Increased concentration of portal GLP-1<br>- Decreased the glycemic response and plasma glucose concentration	- Benefits to glycemic response and fasting plasma glucose in mice<br>- Improves the efficacy of metformin<br>→ The present study proposes a benefit for combining probiotics and/or prebiotics with antidiabetic drugs	(154)	2015
